# Supplementary material for: Complexome profiling of the Chlamydomonas psb28 mutant reveals TEF5 as an early PSII assembly factor
Source: Plant Cell. 2025 Mar 17;37(6):koaf055. doi: 10.1093/plcell/koaf055 (PMC12164590; doi:10.1093/plcell/koaf055)
Supplement: koaf055_Supplementary_Data [file koaf055_supplementary_data.zip › psb28_interactive.zip!/plots/intensity/qualityPlots/peptides/_Cre09.g416800.t1.2.html]

### Cre09.g416800.t1.2

Trivial name:   
SeqCoverage [median]: 16.90  
Mass: 22.78 kDa  
  
 

| Sequence | MissedCleavage | Mass [Da] | wtIntensity | muIntensity | Score |
| --- | --- | --- | --- | --- | --- |
| AATDADLPARR | 1 | 1155.60 | 363.05 | 1203.94 | 90.05 |
| FQYDLSDR | 0 | 1042.47 | 363.36 | 0.00 | 63.48 |
| LGAGLALASSSATPGAK | 0 | 1470.80 | 0.00 | 1248.40 | 68.54 |
|  |  |  | 726.4 | 2452.3 |  |
